# Parent-perceived oral habits among a group of school children: prevalence and predictors

**DOI:** 10.1038/s41405-024-00261-0

**Published:** 2024-10-05

**Authors:** Nagwa Mohamed Ali Khattab, Mennat Allah Ashraf Abd-Elsabour, Ola Moustafa Omar

**Affiliations:** 1 Pediatric Dentistry and Dental Public Health Department, Faculty of Dentistry, Ain Shams University, Cairo, Egypt; 2 Pediatric and Community Dentistry Department, Faculty of Dentistry, Ahram Canadian University, Giza, Egypt; 3 Pediatric Dentistry and Dental Public Health Department, Faculty of Dentistry, Cairo University, Cairo, Egypt

**Keywords:** Dental public health, Preventive dentistry

## Abstract

**Purpose:**

Practicing oral habits beyond the normal age range is assumed to be due to underlying psychological disturbance and could result in a deformation of the orofacial structure. The first step in managing such a health condition is to evaluate its size. Thus, this study aimed primarily to assess the prevalence of oral habits among a group of school children aged from 5 to 7 years, in Cairo, Egypt. The secondary aim of the study was to investigate some possible related predictors along with the mother’s perception of the child’s oral health-related quality of life.

**Methods:**

A Google form questionnaire was designed, utilizing the third domain of Nordic Orofacial Test-Screen (NOT-S), to assess the presence or absence of oral habits and their types, if reported. Also, there were two global rating items to test the child’s oral health-related quality of life from the mother’s prospection, along with one item to inquire if the mother thinks that the oral habits are harmful to the child. A total number of 23 schools in Cairo, Egypt were randomly selected, and the link to the Google form was distributed through the parent’s groups on social media. All high-quality complete responses were analyzed using the SPSS program, and a Log-binomial regression model was constructed, to determine the significant predictors of practicing oral habits in children.

**Results:**

Among the analyzed 1128 responses, the total number of answers to the third domain of interview part of NOT-S was 1235, with a response rate of (60.39%), no habits were reported in 635 children (51.4%), while nail biting was noted in 21.8%, bruxism in 17.9% and Sucking habits in 8.9%. In total, 63.8% of children who were reported by their parents to be the “only child” didn’t practice any habit, and a higher prevalence of oral habits was detected in children with siblings. There was no detectable association between the mother’s educational level and practicing any of the habits, although there was an association between the mother’s educational level and their awareness of the harmful effect of oral habits on the child. The mothers’ answers to the global rating items were not associated with any of the oral habits.

**Conclusion:**

The most prevalent oral habit in the current study was the nail-biting habit. The presence of other siblings and the number of siblings were contributory factors in the occurrence of oral habits, while mothers’ educational level was not associated with practicing oral habits. The mothers’ awareness of the harmful effect of oral habits on the children was not satisfactory, and there was no association between oral health-related quality of life and the children’s oral habits, from the mothers’ perspective.

## Introduction

*Habit* in simple words is the repetition of a certain action, without a real conscious desire [1, 2]*. Deleterious oral habits* (DOH) are defined as “a form of behavior, practiced using orofacial structures, that is picked up due to its frequent repetition without functional use”. Nail biting, finger or object sucking, lip, tongue, or cheek biting, clenching and bruxism, and mouth breathing are all examples of deleterious oral habits [3].

Practicing one or more of these oral habits was proved to have a damaging effect on the orofacial and dental structures, due to exertion of a minute force on the same spot for a long period [3, 4], which consequently compromises the child’s oral health-related quality of life (OHRQoL) [5, 6]. Children who continue practicing oral habits beyond the normal age range are expected to have a malocclusion [7–9] and the need for future orthodontic treatment [4].

The child’s parents are the primary caregivers and the responsible adults for all their life aspects, including their health and well-being [10]. Parents’ educational level, knowledge, and attitude toward oral health influence the child’s oral health to a great extent [11, 12]. Also, their perception of the child’s oral health status, especially during the early years of the child’s life, could be used as a valid measurement for the child’s OHRQoL [13].

It was also proved that there is a strong positive association between practicing oral habits in children beyond the normal age range and underlying psychological disturbance [14–16]. A meta-analysis studying the association and awake bruxism reported a positive association (OR 2.07 [1.51, 2.83], *p* < 0.00001) [15]. It is assumed that the vulnerable child when feels stressed, secures their performance by practicing deleterious oral habits practicing as a coping mechanism [14, 17].

The first step in dealing with any healthcare problem is to know its exact size and effect on the target population. It is recommended by the WHO to plan dental health care services and oral health education programs as per the survey studies [18]. The prevalence of oral habits was investigated in many countries, among children of different age groups, and it was proven to be high worldwide [4, 19–23].

To the best of our knowledge, there are very few studies investigating the prevalence of oral habits among school children in the critical age group of 5–7 years, especially in Cairo, Egypt [5, 17, 24]. None of them aimed to identify the prevalence and determinants of practicing oral habits among those children. In order to establish well-designed preventive, interventional, and educational programs addressing practicing oral habits as a healthcare problem, baseline data regarding the size of the problem and its governing factors is essential. Accordingly, the primary aim of the current study was to assess the prevalence of practicing oral habits among school children aged from 5 to 7 years in Cairo, Egypt. Also, it aims to investigate the association of some possible predictor factors, in addition to exploring the awareness of the mothers about the harmful effect of oral habits on the child, and their perception towards the children’s OHRQoL.

## Subject and methods

### Study design

This study was a descriptive cross-sectional study, conducted during April 2023, using a Google form, that was meant to be filled out by the child’s mother. The research utilized the third domain of Nordic Orofacial Test-Screen (NOT-S) [25] as a validated habit assessment tool. The study followed the methods used in Abd-Elsabour et al. 2023 study [17], with some methodological modifications. Also, there was an administrative chart section to address the number of siblings, child order between siblings, and the mother’s educational level, as possible predictors for practicing oral habits in children. In addition to items to address the child’s OHRQoL from the mother’s perspective, and her knowledge about the harmful effect of practicing oral habits in children. This study is reported per the Strengthening the Reporting of Observational Studies in Epidemiology (STROBE) Statement [26]. Data collection took place during April and May 2023.

### Sample size calculation

According to Darwish 2020 [22]; the prevalence of oral habits among Egyptian children was 29.4%, 15.1%, and 42.1% for tongue thrust, sucking habits, and nail-biting, respectively. The following formula was used for calculating the adequate sample size for this prevalence study [27]:

$${{{\rm{n}}}}={{{\rm{Z}}}}^{2}{{{\rm{P}}}}(1-{{{\rm{P}}}})/{{{\rm{d}}}}^{2}$$

where *n* = sample size, *Z* = *Z* statistic for a level of confidence (1.96 for 95% confidence level), *P* = expected prevalence based on Darwish 2020 [22], *d* = precision (the width of the confidence interval is twice that of the precision).

By adopting the prevalence of the nail-biting habit among Egyptian children (42.1%), which yielded the largest sample, the required sample size was 1041 for an absolute precision of ±3% in estimating the prevalence in the current study with 95% confidence. This sample size is calculated using the Scalex SP calculator [27].

### Participants

Children, reported by their mothers to be healthy, aged from 5 to 7 years were enrolled in this study. The study participants were recruited from 23 primary schools with preschool classes, to match the chosen participants’ age range, in Cairo, Egypt. The random selection of schools was done by a simple randomization technique. A list of all the available primary schools with preschool classes in Cairo, Egypt, was obtained from the local authorities of education (*n* = 1264 schools). Then, the name and address of each school were written on a piece of paper folded eight times, and the piece of paper was put in a sealed opaque envelope. After that, all the sealed opaque envelopes were added to a large container, and an independent researcher, who was not a part of this study, was asked to randomly select 23 envelopes. After the schools’ authorities’ approval, the Google form questionnaire was distributed among schools’ students’ parents’ groups on social media.

### The electronic survey tool

The Google form started with the informed consent section, which explained in detail the study aims and methods, and ended with a phrase denoting that by clicking next, the mother is consenting to participate in the study.

Following a question about “who is filling this questionnaire form?”, the second section was an administrative information collecting section that included the child’s age, sex, number of siblings, child order between their siblings, child’s medical history, and mother’s educational level.

After that, the third section consisted of three questions; “How would you rate the health of your child’s teeth, lips, jaws, and mouth?” [28–30], “How much is your child’s overall well-being affected by the condition of his/her teeth, lips, jaws, or mouth?” [30], and “Do you think that practicing these habits is harmful to the child?”.

The fourth section was designed as a check box tool to investigate the presence or absence of oral habit(s), and its type, using the third domain of the interview part of NOT-S. The options in the check box tool were “the child bites their nails every day”, “the child sucks their fingers or any other object every day”, “the child bites their teeth together hard or grinds their teeth every day”, and “the child does not practice any of the above habits”. A question about the duration of habit(s), if reported, was added to this section. At the end of the Google form, there was an educational file in Arabic, discussing some of the child’s oral health aspects, as a thank-you gift for the study participants. Also, there was an email address provided for any inquiry about oral habits in children.

The translated questions utilized in the third and fourth sections of the survey tool were tested and ensured for understandability and acceptance by interviewing a group of mothers and their children. The first two questions of the third section; “How would you rate the health of your child’s teeth, lips, jaws, and mouth?”, “How much is your child’s overall well-being affected by the condition of his/her teeth, lips, jaws, or mouth?” [30], and “Do you think that practicing these habits is harmful to the child?”, were replicated from the Arabic questionnaire used by Alriamy et al. [31] in their validation study. The third question of the third section of the survey tool, “Do you think that practicing these habits is harmful to the child?”, along with the fourth section were all translated following the recommended method of Beaton et al. [32].

All high-quality complete responses were considered in the analysis, and any response with missing data was excluded, which minimized the potential reporting bias. The study participants were self-selected, which minimized the selection bias, and they filled out the questionnaire by themselves, which omitted the interview bias.

### Statistical analysis

Qualitative data were expressed as numbers and percentages. Quantitative data were presented as mean ± standard deviation (SD) values. Data were explored for normality using the Kolmogorov–Smirnov test and Shapiro–Wilk test, and the results of these tests indicated that data were normally distributed (parametric data), therefore, ANOVA, followed by Bonferroni post hoc test, was used to compare habits. The chi-square test was used for comparisons of qualitative data. The significance level was set at *p* ≤ 0.05.

Also, the Log-binomial regression model was constructed using the prevalence of habits (Yes/No) as the dependent variable while the independent variables were the child’s gender, age, number of siblings, mother’s education, and oral health rating. The regression coefficient (*b*), standard error (SE), and 95% confidence interval, along with the prevalence ratio, were all calculated and reported.

Statistical analysis was performed using a commercially available software program (SPSS 20-Statistical Package for Scientific Studies, SPSS, Inc., Chicago, IL, USA) for Windows.

## Results

**Fig. 1:**
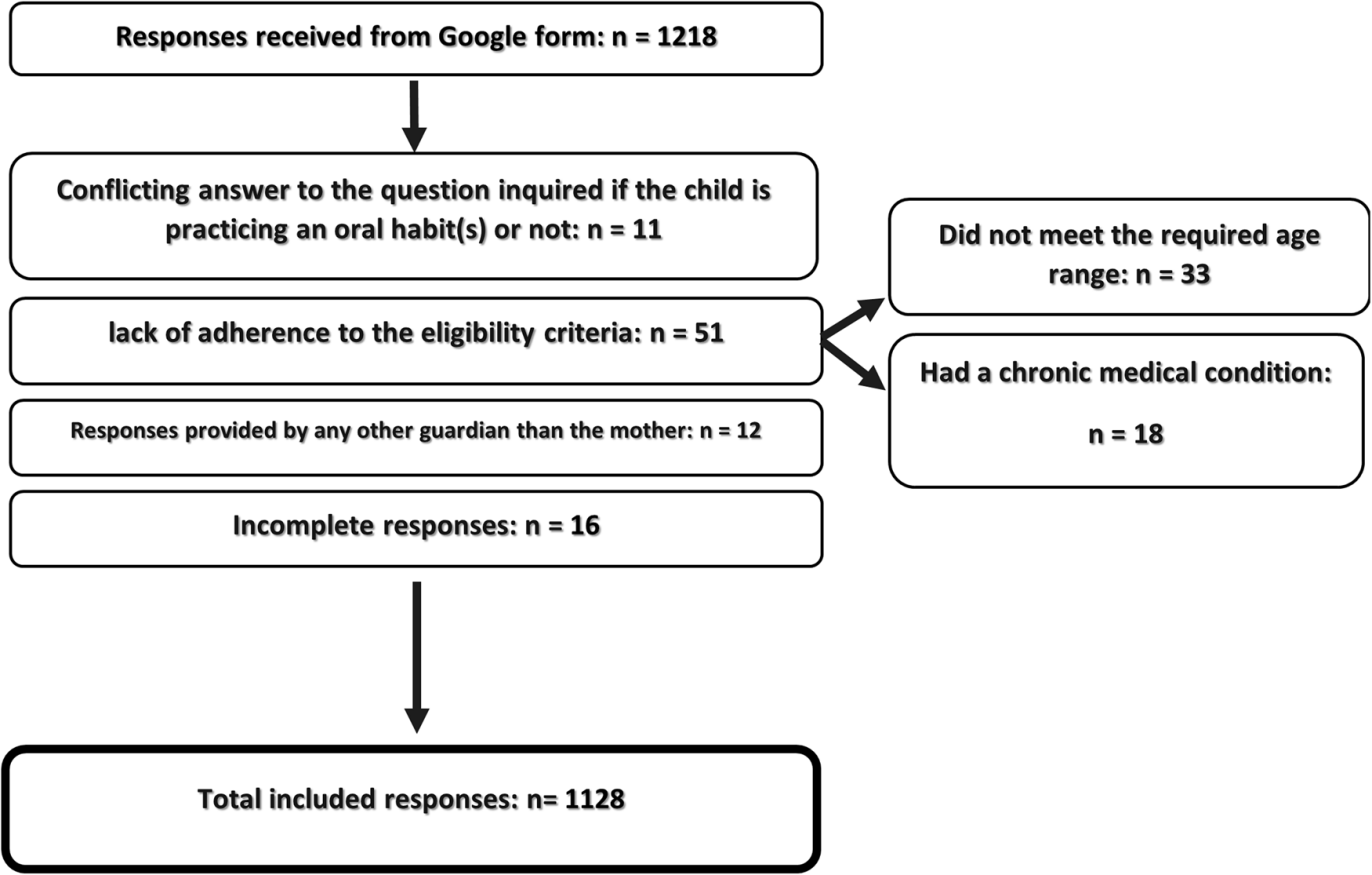
Flow chart illustrating the total number of received responses, numbers of excluded responses, and the reasons for excluding.

The received responses were 1218 out of 2017 targeted school children, with a response rate of 60.39% while the analyzed eligible high-quality completed responses were 1128. Figure 1 is a flow chart illustrating the reasons for excluding 90 responses.

The mean age among the analyzed 1128 responses was 6.01 ± 0.72 years, and there were 576 (51.06%), females, among them there were 328 (56.94%) habit-free females and 248 (43.06%) females practicing one or more oral habits (s), and males 552 (48.94%), among them there were 307 (55.62%) habit-free males and 245 (44.38%) males practicing oral habit(s). Among the study children population, there were 635 (56.29%) children reported to be habit-free, and there were 493 (43.71%) children reported to practice 599 oral habits. There was no significant difference between gender distribution regarding the prevalence of oral habits (*p* = 0.238).

**Fig. 2:**
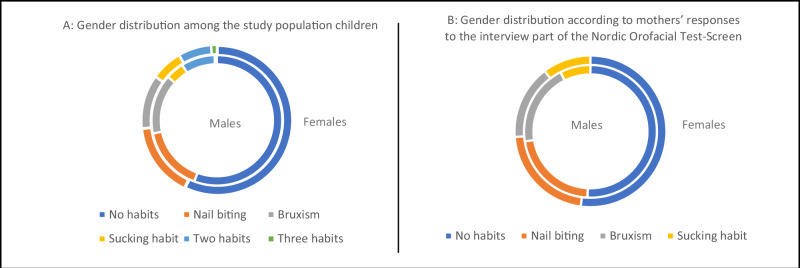
Chart illustrating the prevalence of oral habits and the gender distribution. **A** Gender distribution of the study population of children (the inner circle represents the distribution of males, and the outer circle represents the distribution of females) and **B** gender distribution according to mothers’ responses to the interview part of the Nordic Orofacial Test-Screen (the inner circle represents the distribution of males, and the outer circle represents the distribution of females).

Nail biting was reported in 16.13% of children, bruxism in 13.21%, Sucking habits in 5.76%, two habits in 7.71%, and three habits in 0.89% of children (Table 1 and Fig. 2A).

**Table 1:** Distribution of oral habits among gender.

Gender		Habits				Total	*p* value
		No habits	Nail biting	Bruxism	Sucking habit		
Male	Count	307	132	121	45	605	0.116 ns
	%	24.86%	10.69%	9.80%	3.64%	48.99%	
Female	Count	328	137	100	65	630	
	%	26.56%	11.09%	8.10%	5.26%	51.01%	
Total	Count	635	269	221	110	1235	
	%	51.42%	21.78%	17.89%	8.91%	100.00%	

*ns* non-significant.

Chi-square test.

Significance level *p* ≤ 0.05.

Among a total of 1235 answers to the third domain of interview part of NOT-S, no habits were reported in 51.42%, while nail biting was noted in 21.78%, bruxism in 17.89%, and Sucking habits in 8.91% of children (Table 2 and Fig. 2B).

**Table 2:** Gender distribution according to mothers’ responses to the interview part of the Nordic Orofacial Test-Screen.

Gender		Habits						Total	*p* value
		No habits	Nail biting	Bruxism	Sucking habit	Two habits	Three habits		
Male	Count	307	89	82	25	46	3	552	0.238 ns
	%	27.22%	7.89%	7.27%	2.22%	4.08%	0.27%	48.94%	
Female	Count	328	93	67	40	41	7	576	
	%	29.08%	8.24%	5.94%	3.55%	3.63%	0.62%	51.06%	
Total	Count	635	182	149	65	87	10	1128	
	%	56.29%	16.13%	13.21%	5.76%	7.71%	0.89%	100%	

*ns* non-significant.

Chi-square test.

Significance level *p* ≤ 0.05.

Most children within the study sample were found to have two siblings (*n* = 391). The majority of children practicing no habit, children practicing bruxism, and those who practice sucking habit were reported to also have two siblings (*n* = 219, 55, 26, respectively). The majority of children practicing nail-biting and those who are practicing more than one habit were found to have one sibling (*n* = 63, 45, respectively) (*p* < 0.001) (Table 3).

**Table 3:** Distribution of the number of siblings among the study population children and according to mothers’ responses to the interview part of the Nordic Orofacial Test-Screen.

		Child is the only child	One sibling	Two siblings	Three siblings	Four siblings	*p* value
Number of siblings according to mothers’ responses to the interview part of the Nordic Orofacial Test-Screen	No habits	44	211	219	131	30	*p* < 0.001*
	Nail biting	10	63	59	50	0	
	Bruxism	15	54	55	25	0	
	Sucking habits	0	12	26	22	5	
	Total	69	385	391	243	40	

Chi-square test.

Significance level *p* ≤ 0.05, *significant.

**Fig. 3:**
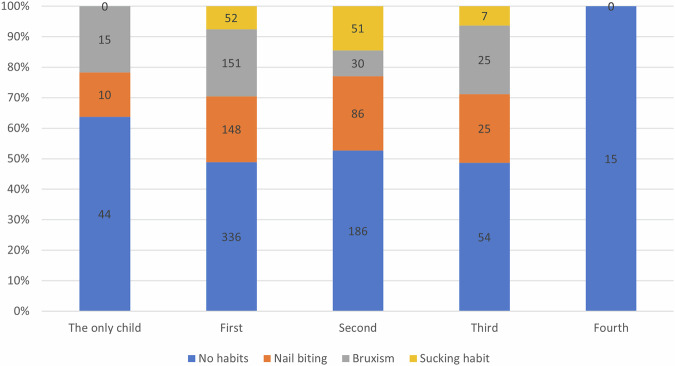
Proportional bar chart illustrating the distribution of oral habits against the child’s order, the first, second, third, fourth, and fifth bars represent the distribution of oral habits among the children who were reported to be the only child, first child, second child, third child, and fourth child, respectively. The number of children in each category is illustrated on the bars.

There was 63.8% of the children who were reported by their parents to be the “only child” didn’t practice any habit, compared to 48.9%, 52.7%, 48.6%, and 100% of the first, second, third, and fourth child respectively, with a statistically significant difference (*p* < 0.001) (Fig. 3).

**Fig. 4:**
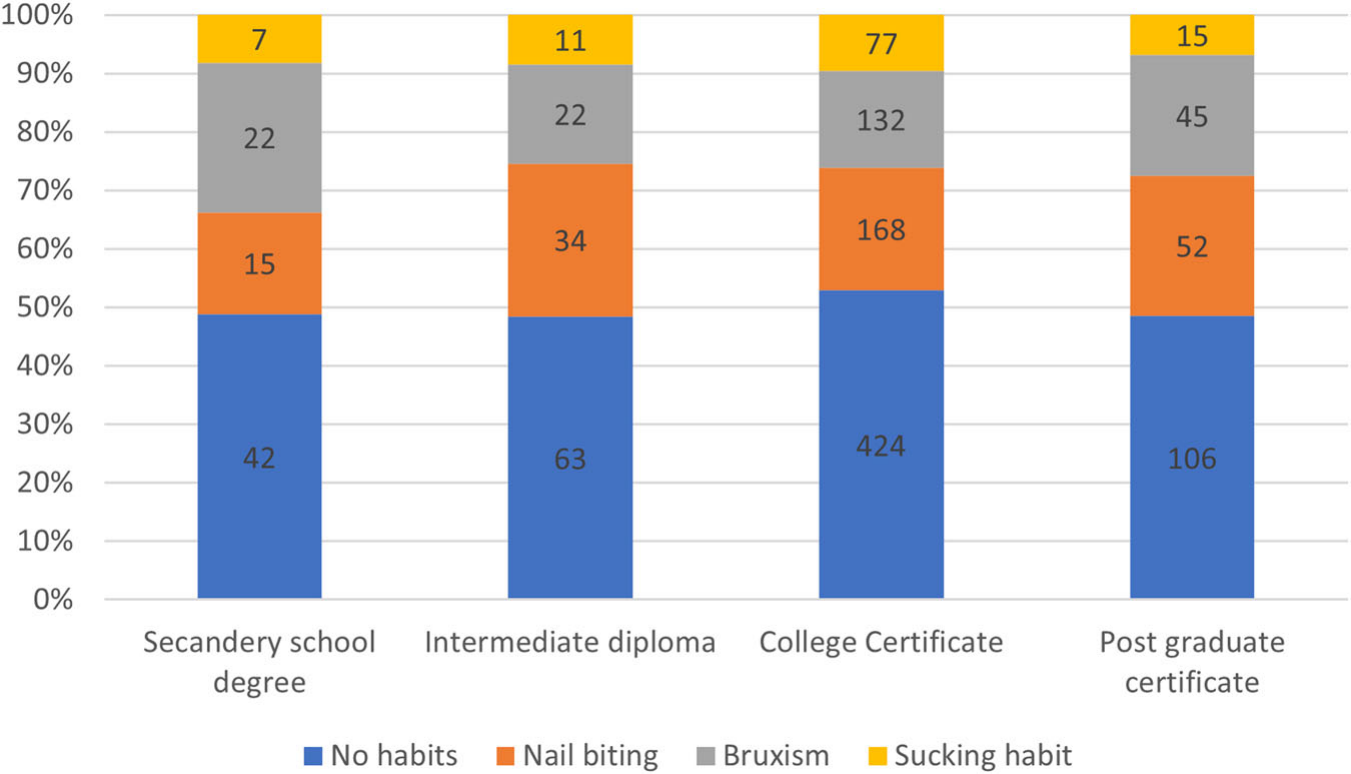
Proportional chart illustrating the distribution of oral habits against the mother’s educational level, the first, second, third, and fourth bars represent the distribution of oral habits among children whose mothers carry a secondary school degree, intermediate diploma, college certificate, and postgraduate certificate, respectively. The number of children in each category is illustrated on the bars.

Also, there was no significant difference between the mother’s education levels regarding the prevalence of oral habits (*p* = 0.364) (Fig. 4).

The mothers’ responses to the three questions in the third part of the Google form are demonstrated in Table 4. There was 71.2% of the mothers holding a post-graduate degree stated that practicing oral habits is harmful to the child, in comparison to only 43.6% of those with a secondary school degree. This difference was statically significant (*p* < 0.001) (Table 5).

**Table 4:** Descriptive statistics of the mothers’ responses to the global rating items and if they think that practicing oral habits is harmful to the child among the study population children

			Habit						Total	*p* value
			No habits	Nail biting	Bruxism	Sucking habit	Two habits	Three habits		
How would you rate the health of your child’s teeth, lips, jaws, and mouth?	Poor	Count	47	6	7	0	5	0	65	*p* < 0.001*
		%	4.17%	0.53%	0.62%	0.00%	0.44%	0.00%	5.76%	
	Fair	Count	73	25	15	10	2	0	125	
		%	6.47%	2.22%	1.33%	0.89%	0.18%	0.00%	11.08%	
	Good	Count	154	50	65	20	32	0	321	
		%	13.65%	4.43%	5.76%	1.77%	2.84%	0.00%	28.46%	
	Very good	Count	270	84	47	25	38	5	469	
		%	23.94%	7.45%	4.17%	2.22%	3.37%	0.44%	41.58%	
	Excellent	Count	91	17	15	10	10	5	148	
		%	8.07%	1.51%	1.33%	0.89%	0.89%	0.44%	13.12%	
	Total	Count	635	182	149	65	87	10	1128	
		%	56.29	16.13	13.21	5.76	7.71	0.89	100.00%	
How much is your child’s overall well-being affected by the condition of their teeth, lips, jaws, or mouth?	Never	Count	287	91	48	34	48	10	518	*p* < 0.001*
		%	25.44%	8.07%	4.26%	3.01%	4.26%	0.89%	45.92%	
	Once or twice	Count	188	50	49	15	14	0	316	
		%	16.67%	4.43%	4.34%	1.33%	1.24%	0.00%	28.01%	
	Sometimes	Count	89	26	9	11	0	0	135	
		%	7.89%	2.30%	0.80%	0.98%	0.00%	0.00%	11.97%	
	Often	Count	59	14	41	5	20	0	139	
		%	5.23%	1.24%	3.63%	0.44%	1.77%	0.00%	12.32%	
	Almost every day	Count	12	1	2	0	5	0	20	
		%	1.06%	0.09%	0.18%	0.00%	0.44%	0.00%	1.77%	
	Total	Count	635	182	149	65	87	10	1128	
		%	56.29	16.13	13.21	5.76	7.71	0.89	100.00%	
Do you think that practicing these habits is harmful to the child?	No	Count	284	59	49	19	28	3	442	0.001*
		%	25.18%	5.23%	4.34%	1.68%	2.48%	0.27%	39.18%	
	Yes	Count	351	123	100	46	59	7	686	
		%	31.12%	10.90%	8.87%	4.08%	5.23%	0.62%	60.82%	
	Total	Count	635	182	149	65	87	10	1128	
		%	56.29	16.13	13.21	5.76	7.71	0.89	100.00%	

Chi-square test.

Significance level *p* ≤ 0.05, *significant.

**Table 5:** Distribution of mothers’ responses to the question “Do you think that practicing these habits is harmful to the child?” according to their educational level.

			Mother’s education				Total	*p* value
			Secondary school degree	Intermediate diploma	College degree	Post-graduate degree		
Do you think that practicing these habits is harmful to the child?	No	Count	44	59	282	57	442	*p* < 0.001*
		%	3.90%	5.23%	25.00%	5.05%	39.18%	
	Yes	Count	34	60	451	141	686	
		%	3.01%	5.32%	39.98%	12.50%	60.82%	

Chi-square test.

Significance level *p* ≤ 0.05, *significant.

Log-binomial regression model revealed that the child’s age was the only statistically significant predictor of practicing oral habits. The prevalence ratio of practicing oral habits among children aged 5 years old is 1.319 times higher than the risk of practicing oral habits among older children (Table 6).

**Table 6:** Log-binomial regression model to detect significant predictors for practicing oral habits in children

The independent variable (predictor)	Regression coefficients (*b*)	Stander error (SE)	*p* value	Prevalence ratio (RR)	95% C.I. for (RR)	
					Lower	Upper
Intercept	−1.484	0.430	0.001*	0.227	0.098	0.527
[Child Age = 5.00 years]	0.277	0.129	0.032*	1.319	1.023	1.699
[Child Age = 5.50 years]	0.013	0.193	0.947	1.013	0.694	1.478
[Child Age = 6.00 years]	−0.151	0.137	0.271	0.860	0.657	1.125
[Child Age = 6.50 years]	0.185	0.170	0.276	1.203	0.862	1.677
[Child Age = 7.00 years]	0			1.000		
[Gender = Female]	0.032	0.093	0.728	1.033	0.861	1.239
[Gender = Male]	0			1.000		
[Siblings = Child is the only child]	0.323	0.447	0.470	1.381	0.575	3.317
[Siblings = one sibling]	0.510	0.394	0.196	1.666	0.769	3.609
[Siblings = two siblings]	0.578	0.396	0.144	1.782	0.820	3.872
[Siblings = three siblings]	0.593	0.399	0.138	1.809	0.827	3.959
[Siblings = four siblings]	0			1.000		
[Mother Education = Secondary school degree]	0.018	0.206	0.931	1.018	0.679	1.526
[Mother Education = Intermediate diploma]	0.028	0.179	0.875	1.028	0.725	1.460
[Mother Education = College degree]	-0.045	0.126	0.722	0.956	0.746	1.225
[Mother Education = post-graduate degree]	0			1.000		
[Oral Health = Never]	−0.413	0.317	0.193	0.662	0.355	1.232
[Oral Health = Once or twice]	0.132	0.206	0.522	1.141	0.761	1.711
[Oral Health = Sometimes]	0.237	0.170	0.163	1.268	0.908	1.769
[Oral Health = Often]	0.051	0.168	0.761	1.052	0.757	1.463
[Oral Health = Almost every day]	0			1.000		

Significance level *p* ≤ 0.05, *significant.

## Discussion

Oral habits prevalence has increased worldwide [19–21, 33] and they have a deleterious effect on orofacial and dentofacial structures, which consequently compromises the child’s quality of life [5, 6] and increases their demand for orthodontic treatment [7–9].

In this study, the mothers were targeted as they expected to best know about their young children’s habit(s), and their perception towards their child’s oral health is expected to be valid [13]. Also, their knowledge about the harmful effects of deleterious oral habits is the key element in intercepting to omit them [10–12]. The utilized Google form questionnaire allowed the recruitment of the required sample of children’s mothers, and ensured a reliable random sampling procedure, without any interview bias.

The age range of the children in the current study was chosen based on the reversible effect age limit, which was reported to be 3–4 years [34, 35]. It was hypothesized that the continuation of practicing deleterious oral habits beyond this age limit would result in deleterious deformation of the dental and orofacial structures [4, 5]. Thus, this study selected the critical age range of 5–7 years. As the chosen age range of the children is younger than being able to answer the Google form questionnaire by themselves, this study targeted the children’s mothers, as they expected to best know about their children, especially at this young age [13].

The third domain of the interview part of NOT-S [25] is a valid method for assessing the oral habits in children in the study age range, and it’s allowed to be answered by the parents when assessing young children [25]. The global rating questions utilized in this study, in addition to being simple and taking a few minutes to answer [29, 36], were proven to be valid in assigning a child’s oral health-related quality of life when answered by the parent [30, 31].

The current study results revealed that the most prevalent practiced oral habit was the nail-biting habit followed by the bruxism habit, and the sucking habit was reported to be the least prevalent habit. This could be justified by the chosen age range, as the development of oral habits in children is divided into three periods; the sucking period, which ends at the age of three, then a transitional period, and next the biting period. The biting period, on which the child develops the nail-biting habit starts at the age of 4–5 years and reaches its peak at 6–12 years [35]. These results go in agreement with the previous study that was carried out in Alexandria, Egypt on school children aged from 6–12 years which reported that the prevalence of nail-biting, and sucking habits was (41.07%), and (15.1%), respectively [22]. On the other hand, the current study results contradict the results reported by Farrag and Awad 2016; which revealed that the prevalence of digit-sucking, nail-biting, and bruxism were (6.1%), (9.7%), and (3.1%), respectively, among school children aged from 6–12 years in Dakahlia governorate, Egypt. Alexandria governorate and Cairo are both considered urban cities with higher socioeconomic levels, unlike Dakahlia city, which is considered a rural city. It is assumed that oral habit prevalence is higher among children in urban cities in contrast with rural cities [33]. The study findings also go in agreement with the study carried out by Aloumi et al.; in Saudi Arabia on children aged from 3–6 years which revealed that the most practiced habit was the nail-biting habit (27.2%), followed by the thumb sucking habit (7.4%) and the least prevalent habit was the teeth clenching habit (6.0%) [21].

Also, this finding agrees with the study carried out by Rai et al.; on Nepal children aged from 3 to 7 years which reported that the most prevalent habit was Nail biting (19.5%), followed by bruxism (16.9%), while the least prevalent habit was the sucking habit (14.3%) [20]. On the other hand, the present results disagreed with Kolawole et al. [19]; who reported that the most prevalent oral habit among Nigerian children aged from 1 to 12 years(s) was the sucking habits (50%), followed by nail-biting (11.2%) and then the bruxism habit (9.8%). This disagreement between the current study and Kolawole et al.; could be explained by the wide age range in the previous studies.

Regarding gender predilection, it was noted that the prevalence of sucking habits among females was higher than among males, in contrast to the bruxism habit, which was more practiced by males, while the nail-biting habit showed no sex differences. These results go in accordance with Maia-Nader et al. [33]; who reported that the female gender is one of the predictive factors for non-nutritive sucking habits, and Rai et al. [20]; who reported that bruxism was practiced by males more than females. This could be justified by the fact that females could be affected more than males by emotional disturbance, and they are more open to seeking emotional security through sucking habits [33], while males tend to avoid emotional processing, and focus externally, which makes them more susceptible to practice the bruxism habit [37]. Also, females were found to have a higher prevalence risk of practicing oral habits when compared to males.

It was observed that a higher number of siblings significantly influenced practicing sucking habits, whereas a higher percentage of children practicing sucking habits belonged to larger size families. Also, it is worth mentioning that the majority of the children who were reported to be “the only child” did not practice any oral habits. This could be justified by the attachment theory which states that the child thrives to build an emotional bond with a responsive caregiver and demand their attention and affection. The arrival of a new sibling will consume most of the caregiver’s attention and affection, which will drive the older child into separation anxiety, and if not managed properly by the caregiver, will lead to emotional disturbance, which the child may express in the form of oral habit(s), as an attempt to obtain emotional security [38]. This could be augmented by the results of the regression analysis which revealed that being the only child has a lower prevalence ratio of practicing oral habits compared to having three siblings (1.38, and 1.80, respectively).

There was no association between the mothers’ educational levels and practicing oral habits in children in the current study, and the level of mother’s education was an insignificant predictor of practicing oral habits with a prevalence risk nearly equal to one in all levels of mother’s education. This is an observation that was confirmed by Maia-Nade et al. [33]; who found in their population-based cohort study that there was no association between maternal education and practicing non-nutritive sucking habits among children in both developed and underdeveloped cities in Brazil.

The mother’s answers “never” to the global rating question “How much is your child’s overall well-being affected by the condition of their teeth, lips, jaws, or mouth?” were a negative predictor of practicing oral habits in children. Also, most mothers answered the question “How would you rate the health of your child’s teeth, lips, jaws, and mouth?” with “very good” had a child who does not practice oral habits. This result goes as per a previous cohort study conducted in Cairo, Egypt (Abd-Elsabour et al. [17]) among children of the same age group and reported that the OHRQoL of the habit-practicing group perception was 1.5 times worse than that of the habit-free group parents’ perception. The selected age range in the current study was similar to that selected in Abd-Elsabour et al. [17]; study and the parents were targeted as proxies to answer the quality-of-life items in both studies. Also, the current results go in agreement with Leme et al. [6]; who reported a higher mean score of oral health-related quality of life questionnaire among both groups of habit-practicing children aged from 8 to 10 years, and 11 to 14 years, compared to children who do not practice oral habit. However, Leme et al. selected an older age range and targeted the children themselves to answer the OHRQoL items, and obtained the same result obtained in the current study.

In the present study, the percentage of mothers who answered the question “Do you think that practicing oral habits is harmful to the child?” correctly was not satisfactory and reflects the demand for incorporation of the oral habit(s) in the oral health educational programs. This finding goes in accordance with Alves et al. [39]; who reported insufficient parent/caregiver knowledge about sleep bruxism in their children, which hinders the parent from seeking help to prevent such habit, and consequently worsens the condition and exaggerates its harmful effect [39].

The current results revealed that a higher mother’s educational level was associated with a higher percentage of correct answers, which confirms the effect of the mother’s education on improving her oral health knowledge, science well-educated mothers have better access to information related to childcare, and consequently, they are more aware of many common oral health issues that might affect the child [11].

It was also noted that the percentage of parents who answered this question correctly was lower (55.3%) among children who do not practice oral habit(s) than those of the children practicing oral habit(s) (67.6%, 67.1%, 70.77%, 67.8%, and 70%, for different habit groups), the same finding was reported by Alves et al. [39]; as they reported in their study that the percentage of parents of bruxer children answered the question “Dose bruxism affect health” correctly (84.6%) was higher than that of parents of the non-bruxer children (67.5%). This could be justified by the fact that the parent is more aware of the condition’s deleterious effect when his child practices it.

The only significant predictor in this study was age, with a prevalence risk of practicing oral habits is 1.3 when the child is 5 years. this is justified by the fact that young children tend are easily undergo a psychological disturbance and tend to internalize the surrounding problems which makes them more vulnerable to stressors [2, 40, 41].

The applicability of this study’s results is dependent on many factors, as oral habits are influenced by many variables as the socioeconomic level, the resilience of the child’s personality, the strength of the child-caregiver relationship, the child’s age range, sex, number of siblings, their order between their siblings, and many other factors. Thus, each population is expected to have its own findings in accordance with these variations.

According to the results of the current study, it is recommended to design preventive and educational programs, that are proportional to the size of children practicing oral habits obtained in this study. This when done as early as possible in the child’s life, would save much cost of treating the dental, skeletal, and psychological effects later in his life. It is also recommended that children practicing more than one habit receive special attention, and further studies are recommended to address the effects and determinants of this condition.

Although this study was limited by the lack of clinical examination and assessment of the severity of habit and its impact on oral health, susceptibility to non-respondent bias due to the moderate responding rate, it provided baseline records that could be beneficial in both future similar studies as well as planned educational and preventive programs. These records were obtained using NOT-S as a valid measure of oral habits. It also provided evidence for the importance of drawing the parents’ attention to the compromising effect of these habits on the child’s OHRQoL.

## Conclusions

The most prevalent oral habit in the current study was the nail-biting habit. The presence of other siblings and the number of siblings were contributory factors, while mothers’ educational level was not associated with practicing oral habits. The mothers’ awareness of the harmful effect of oral habits on their children was not satisfactory, and there was no association between OHRQoL and the children’s oral habits, from the mother’s point of view.

## Data Availability

Raw data (master table) is available upon request from the corresponding author through email (mennatallah.ashraf@dentistry.cu.edu.eg).
